# Warming Affects Growth Rates and Microcystin Production in Tropical Bloom-Forming *Microcystis* Strains

**DOI:** 10.3390/toxins10030123

**Published:** 2018-03-14

**Authors:** Trung Bui, Thanh-Son Dao, Truong-Giang Vo, Miquel Lürling

**Affiliations:** 1 Aquatic Ecology & Water Quality Management Group, Wageningen University, P.O. Box 47, 6700 AA Wageningen, The Netherlands; miquel.lurling@wur.nl; 2 Institute for Environment and Resources, Vietnam National University, Hochiminh City, Linh Trung Ward, Thu Duc District, 700000 Ho Chi Minh City, Vietnam; 3 Hochiminh City University of Technology, Vietnam National University, Hochiminh City, 268 Ly Thuong Kiet Street, District 10, 700000 Ho Chi Minh City, Vietnam; dao.son@hcmut.edu.vn; 4 National Breeding Center for Southern Marine Aquaculture, 167 Thuy Van Street, Vung Tau Town, Ba Ria 790000, Vung Tau Province, Vietnam; truonggiang_9793@yahoo.com.vn; 5 Department of Aquatic Ecology, Netherlands Institute of Ecology (NIOO-KNAW), P.O. Box 50, 6700 AB Wageningen, The Netherlands

**Keywords:** cell quota, climate change, cyanobacterial blooms, cyanotoxins, Mekong delta, Vietnam

## Abstract

Warming climate is predicted to promote cyanobacterial blooms but the toxicity of cyanobacteria under global warming is less well studied. We tested the hypothesis that raising temperature may lead to increased growth rates but to decreased microcystin (MC) production in tropical *Microcystis* strains. To this end, six *Microcystis* strains were isolated from different water bodies in Southern Vietnam. They were grown in triplicate at 27 °C (low), 31 °C (medium), 35 °C (high) and 37 °C (extreme). Chlorophyll-a-, particle- and MC concentrations as well as dry-weights were determined. All strains yielded higher biomass in terms of chlorophyll-a concentration and dry-weight at 31 °C compared to 27 °C and then either stabilised, slightly increased or declined with higher temperature. Five strains easily grew at 37 °C but one could not survive at 37 °C. When temperature was increased from 27 °C to 37 °C total MC concentration decreased by 35% in strains with MC-LR as the dominant variant and by 94% in strains with MC-RR. MC quota expressed per particle, per unit chlorophyll-a and per unit dry-weight significantly declined with higher temperatures. This study shows that warming can prompt the growth of some tropical *Microcystis* strains but that these strains become less toxic.

## 1. Introduction

Globally more frequent and intense cyanobacterial blooms are occurring [1,2,3] with climate change being viewed as the most plausible cause [4,5]. Both direct warming effects on growth and indirect effects on water column stability are expected to promote cyanobacteria over freshwater eukaryotic phytoplankton at elevated temperatures [4,5,6,7]. Moreover, synergism between temperature and nutrients in eutrophic and hyper-eutrophic lakes [8,9] and warming-enhanced nutrient loading with rising temperatures are also predicted to intensify cyanobacterial blooms [3,5,10,11]. 

Such blooms may come with risks for the aquatic ecosystem causing anoxia related fish kills and food web alterations [12,13]. Cyanobacterial blooms may be a threat to animal and public safety as many cyanobacteria produce a variety of potent toxins [14,15]. For instance, 458 suspected human illnesses and 175 animal deaths have been linked with bloom events in the U.S.A. during 2007–2011 [16]. Cyanobacteria can produce a variety of toxins of which the microcystins (MCs) are most abundant globally. The main producer of MCs is the cosmopolitan cyanobacterium *Microcystis* [17]. Despite a wealth of information in the literature on cyanobacterial blooms and their toxins [18], consequences for the toxicity of cyanobacteria under global warming are less well studied. Laboratory experiments [19,20] as well as field observations [21,22] suggest that toxic strains in a bloom could be benefitting more from warming than the non-toxic ones, yet not always correlations with temperature are found [23]. Nonetheless, toxin quota likely decrease with higher temperatures as has been reported for MC producing organisms based on toxicity measurements [24,25] and direct quantification of the toxin quota [26,27,28,29,30]. The information for tropical strains is even more limited [28] and to date no indication exist on temperature affected toxicity of cyanobacteria in Southern Vietnam. 

Southern Vietnam, including the Mekong Delta, is expected to be one of the most extremely impacted regions by climate change [31,32]. This region is the food basket of Vietnam with ongoing rapid development of intensive agriculture and aquaculture, which are main sources of pollution through discharging pesticides [33] and nutrients [34]. However, there is a clear knowledge gap on occurrence, dominance, distribution, toxins and toxicity of cyanobacteria in Southern Vietnam. A few studies have focused on toxin producing cyanobacteria in natural lakes and reservoirs in Vietnam [35,36,37], of which Pham et al. [38] detected MC at maximally 2.5 µg L^−1^ in Dau Tieng Reservoir. The heat waves in the Indochina peninsula in recent years [39] may be an indication of near future conditions. To get more insight in how one of the most abundant cyanobacteria, *Microcystis* [17] will respond to different temperatures, we isolated six *Microcystis* strains from fish ponds and a reservoir in South Vietnam to test the hypothesis that temperature would affect growth rates and that MC cell quota would decline with rising temperature. 

## 2. Results

### 2.1. Water Bodies in Which Microcystis Strains Were Isolated

**Figure 1:**
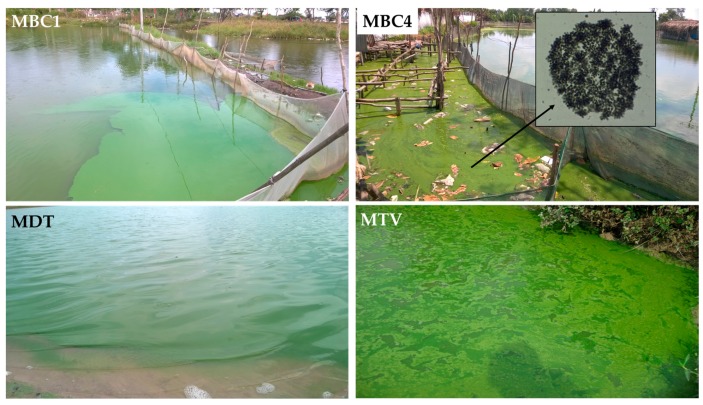
Photographs of cyanobacteria accumulations in three fish ponds (MBC1, MBC4 and MTV) and a reservoir (MDT). Microscopy revealed that Microcystis was a dominant bloom former in all sampled accumulations.

Six strains of *Microcystis* were isolated from six different surface waters (see Section 5.1 for more details). Strains MBC1, MBC2, MBC3 and MBC4 came from four fish ponds in BinhChanh district (HoChiMinh city, Vietnam), strain MDT was from DauTieng reservoir (TayNinh, Vietnam) and strain MTV was from a fish pond in the Mekong delta (TraVinh, Vietnam). At the moment of sampling all six surface waters were characterised by high water temperatures, relatively high pH and high total nitrogen (TN) and total phosphorus (TP) concentrations that indicated the waters were hyper-eutrophic [40] (Table 1). Water temperatures ranged from 33.1 to 37.7 °C at the sampling sites where cyanobacterial blooms were observed (Figure 1). Samples of these dense cyanobacteria accumulations showed high chlorophyll-a concentrations (Table 1). Microscopic inspection revealed that blooms were dominated by *Microcystis* (Figure 1).

### 2.2. Effects of Temperature on Microcystis Biomass Indicators 

**Figure 2:**
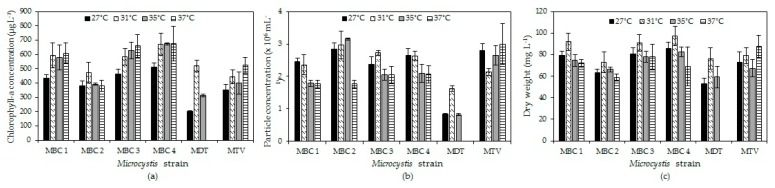
Chlorophyll-a concentrations (**a**), particle concentrations (**b**) and dry-weight (**c**) of six *Microcystis* cultures after six days culturing at four different temperatures. Error bars indicate 1 SD (*n* = 3).

In four strains (MBC1, MBC3, MBC4, MTV) chlorophyll-a concentrations increased with temperature, in one strain (MBC2) there was hardly a difference, while in strain MDT chlorophyll-a concentration increased at 31 °C and declined to become zero at 37 °C (Figure 2a). A two-way ANOVA indicated a significant temperature effect (*F*_3,71_ = 23.4; *p* < 0.001), a significant strain effect (*F*_5,71_ = 69.9; *p* < 0.001) and a significant strain x temperature interaction (*F*_15,71_ = 8.9; *p* < 0.001). This interaction effect was due to the deviating response of strain MDT that died at 37°C. Omitting this strain from the analysis still yielded a significant temperature effect (*F*_3,59_ = 15.9; *p* < 0.001), a significant strain effect (*F*_4,59_ = 30.0; *p* < 0.001) but no strain x temperature interaction (*F*_12,59_ = 1.5; *p* = 0.179). Tukey’s test revealed three homogenous strain groups: (1) MBC2 and MTV, (2) MBC1 and MBC3, (3) MBC3 and MBC4.

For particle concentrations, no two-way ANOVA could be performed due to non-normality and unequal variances in the data but the patterns observed were similar to those for chlorophyll-a and dry-weight (Figure 2b).

In all strains, dry-weight was higher at 31 °C than at 27 °C and then dry-weights slight declined with higher temperatures in strains MBC1-4, strongly in strain MDT, or slightly increased in strain MTV (Figure 2c). A two-way ANOVA on dry-weights indicated a significant temperature effect (*F*_3,71_ = 27.1; *p* < 0.001), a significant strain effect (*F*_5,71_ = 37.0; *p* < 0.001) and a significant strain x temperature interaction (*F*_15,71_ = 8.4; *p* < 0.001). This interaction effect was mostly due to the deviating response of strain MDT and mitting this strain from the analysis almost completely lifted the strain x temperature interaction (*F*_12,59_ = 2.0; *p* = 0.046). 

### 2.3. Effects of Temperature on Microcystis Growth Rates 

Rising temperature had different, strain depended effects on the growth rates of *Microcystis* strains (Table 2). Since data transformation could not solve non-normality, the effect of temperature on growth rates was tested for each strain separately by one-way ANOVA instead of a two-way ANOVA over all data per endpoint (chlorophyll, particle- and bio volume concentration). In strains MBC1, MBC3 and MBC4 chlorophyll-a based growth rates were significantly higher at 31, 35 and 37 °C than at 27 °C; in strain MBC2 only growth rates at 31 and 35 °C were higher, in strain MTV growth rates remained similar, while in strain MDT growth was significantly promoted at 31 °C but sharply declined at 35 °C and all cells had died at 37 °C after 4 days exposure (Table 2). 

Particle concentration based growth rates showed significantly higher growth rates at higher temperatures for strains MBC1, MBC3 and MBC4, no effect for strain MBC2, significantly higher growth at 35 °C in strain MTV and a similar pattern as for chlorophyll-a based growth in strain MDT where growth was significantly promoted at 31 °C, sharply declined at 35 °C and all cells had died at 37 °C after 4 days exposure (Table 2). Biovolume based growth rates showed a little more within group variability, which caused for instance no statistically significant differences in strain MTV despite growth rates at 37 °C were almost 50% higher than at 27 °C (Table 2). 

Taking for each strain the temperature at which it showed the highest growth rate yielded that the highest overall chlorophyll based growth rates were found at an average temperature of 34.3 °C (±2.7 °C), particle based growth at 33.7 °C (±2.1 °C) and bio volume based growth at 35.0 °C (±2.2 °C), which all three were statistically similar (*H*_2_ = 1.43; *p* = 0.490).

### 2.4. Effects of Temperature on Microcystins

**Figure 3:**
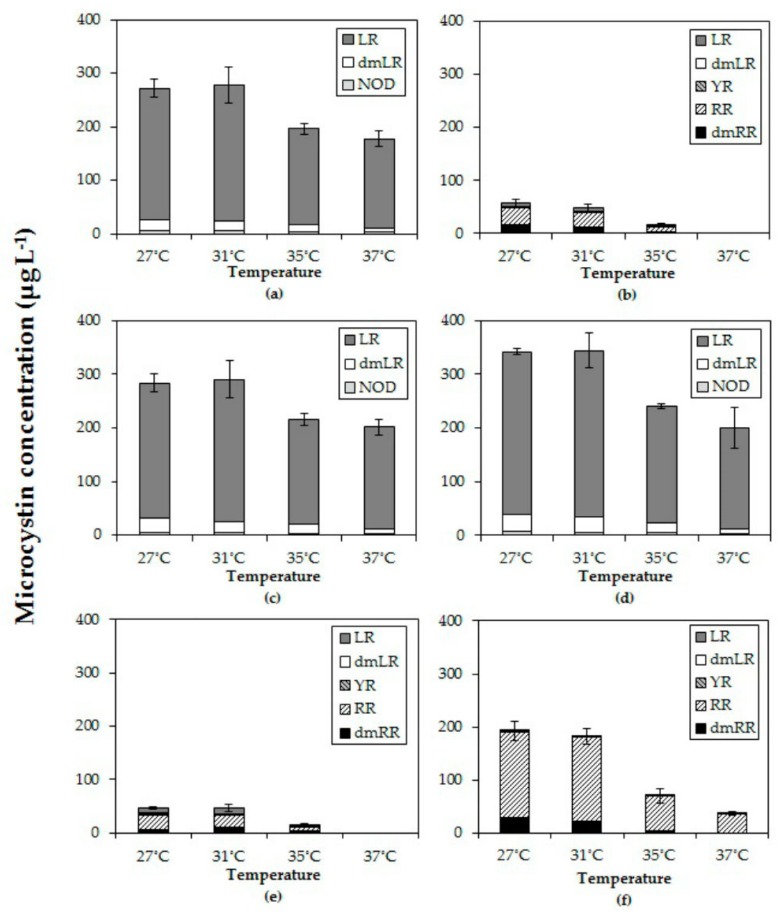
Total microcystin (MC) concentrations (µg/L) and the MC variants in six different *Microcystis* strains grown at four different temperatures (**a**) MBC1, (**b**) MBC2, (**c**) MBC3, (**d**) MBC4, (**e**) MDT, (**f**) MTV.

**Figure A1:**
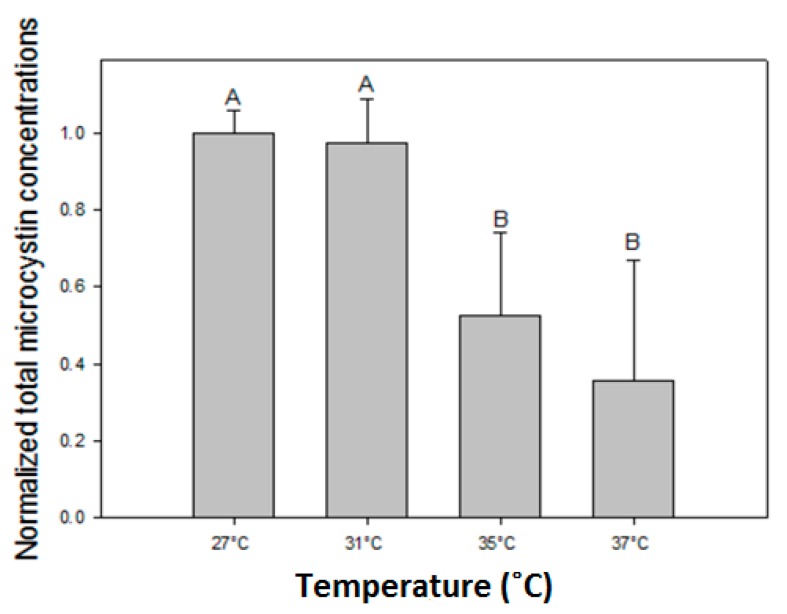
Normalized microcystin (MC) concentrations where the MC concentration in each incubation for each of six strains is normalized against the mean MC concentration at 27 °C. Error bars indicate one standard deviation (*n* = 12). Similar letters (A, B) above the bars represent homogeneous groups that are similar (Tukey test).

All six *Microcystis* strains produced MC with different variants consisting of LR, dmLR in strains MBC1, 3 and 4; LR, dmLR, YR, RR, dmRR in MBC2, MDT and MTV. Some minor quantities of a NOD were detected in MBC1, 3 and 4. The total MC concentration per mL of culture in all six strains declined with higher temperatures (Figure 3). In fact, in strains MBC2 and MDT no MCs were detected anymore at 37 °C. In each strain, total MC concentration was significantly lower at the highest temperature than at the lowest (Table 3). Strains with a comparable MC profile also responded similarly to temperature (Figure 3); strains MBC1, MBC 3 and MBC4 with predominantly MC-LR had two homogeneous groups (27 °C, 31 °C and 35 °C, 37 °C). To evaluate an overall response to different temperatures the MC concentrations for each strain were normalized against the mean MC concentration determined at 27 °C. The overall pattern clearly revealed a significant decline in MC concentrations at higher temperatures (Appendix A, Figure A1).

**Figure A2:**
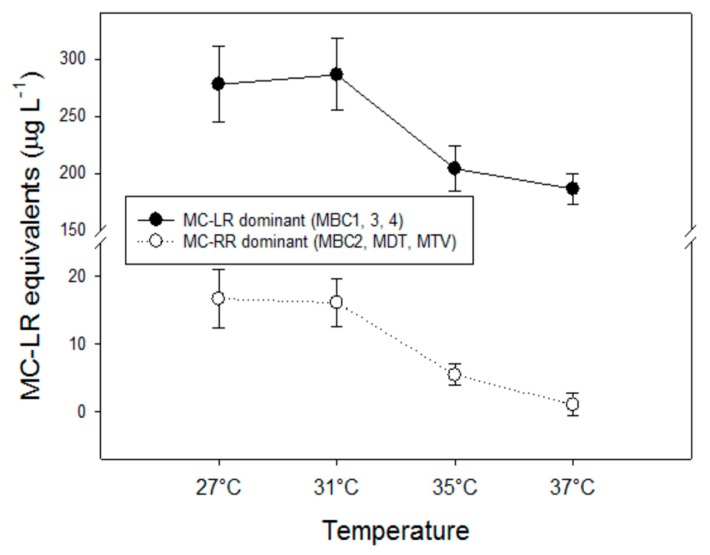
MC-LR equivalents for two groups of strains (the more toxic MC-LR dominated ones, MBC1, MBC3, MBC4 and the less toxic MC-RR dominated ones, MBC2, MDT, MTV) at four temperatures. Error bars indicate one standard deviation (*n* = 3; mean of each strain).

MC-LR was found in all strains and it was the most abundant in MBC1, MBC3 and MBC4 that also contained about 10% dmMC-LR and a trace of an unknown NOD (Figure 3). MC-RR was dominant in the other three strains (MBC2, MDT and MTV) with dmMC-RR as second most dominant MC variant; these strains also contained MC-YR, dmMC-LR and MC-LR (Figure 3). None of the strains contained detectable traces of hydrophobic MCs (MC-LY, MC-LW, MC-LF). Inasmuch as different variants may exert different toxicity, the toxicity expressed in MC-LR equivalents was estimated using toxicity conversion factors for each variant. Combining these for the three MC-LR dominated strains and for the three MC-RR dominated strains clearly showed that toxicity of the MC-LR dominated strains was much higher than that of the MC-RR dominated ones (Appendix B, Figure A2). In both groups toxicity dropped with temperature (Appendix B).

**Figure A3:**
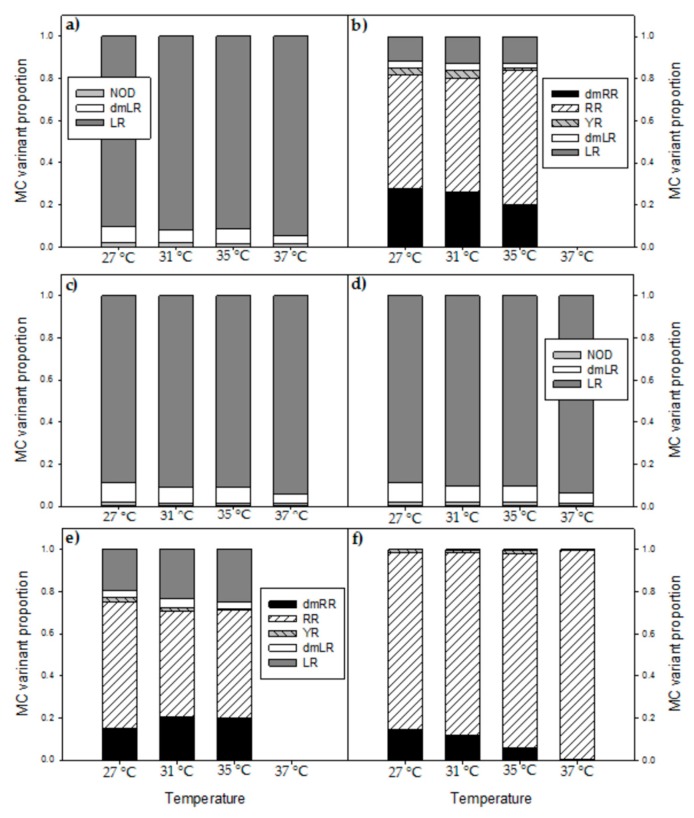
Proportions of different MC-variants and NOD detected in the six strains from Southern Vietnam after six days growth at four different temperatures. (**a**) MBC1, (**b**) MBC2, (**c**) MBC3, (**d**) MBC4, (**e**) MDT, (**f**) MTV.

In all three strains in which MC-LR was most abundant (MBC1, MBC3 and MBC4) the relative abundances changed in a comparable way with temperature (Appendix C, Figure A3). The proportion of MC-LR increased from 89 % (±1%) of the total MC pool in strains grown at 27 °C to 94% (±1%) in strains grown at 37 °C (Appendix C, Figure A3). Holm-Sidak post hoc comparisons revealed three homogeneous groups in each strain: (1) the MC-LR proportion at 27 °C, (2) the MC-LR proportions at 31 °C and 35 °C and (3) the MC-LR proportion at 37 °C (Appendix C; Table A1). The variant dmLR decreased from 9% at 27 °C to 7% at 31 °C and 35 °C and further to 4% at 37 °C in these three strains (Appendix C, Figure A3). Also, a trace of NOD was detected. 

In the three other strains (MBC2, MDT, MTV) MC-LR was far less abundant (Figure A3). In strain MBC2 MC-LR made up 12% (±1%) of the MC-pool at 27 °C, 31 °C and 35 °C but no MC-LR was detected anymore at 37 °C. In strain MDT MC-LR comprised 19% (±2%) of the MC-pool at 27 °C, which increased significantly to 24% (±1%) and 25% (±1%) of the MC-pool at 31 °C and 35 °C, respectively. In strain MTV, no differences were found in the minor proportions of MC-LR (0.2% ± 0.2%) detected at the four incubation temperatures.

The proportion of MC-RR in strain MBC2 was similar at 27 °C and 31 °C making up 54% (±1%) of the MC-pool but significantly elevated at 35 °C making up 64 % (± 2%) of the MC-pool (Appendix C). In strain MDT the opposite was observed, MC-RR comprised 60% (±2%) of the MC-pool at 27 °C, which was significantly more than the 51% (±1%) at 31 °C and 35 °C. In strain MTV, the proportion of MC-RR increased from 84% (±1%) at 27 °C to 87% (±1%) at 31 °C and further to 93% (±5%) at 35 °C and to 99% (±1%) at 37 °C (Appendix C).

### 2.5. Microcystin Cell Quota

**Figure 4:**
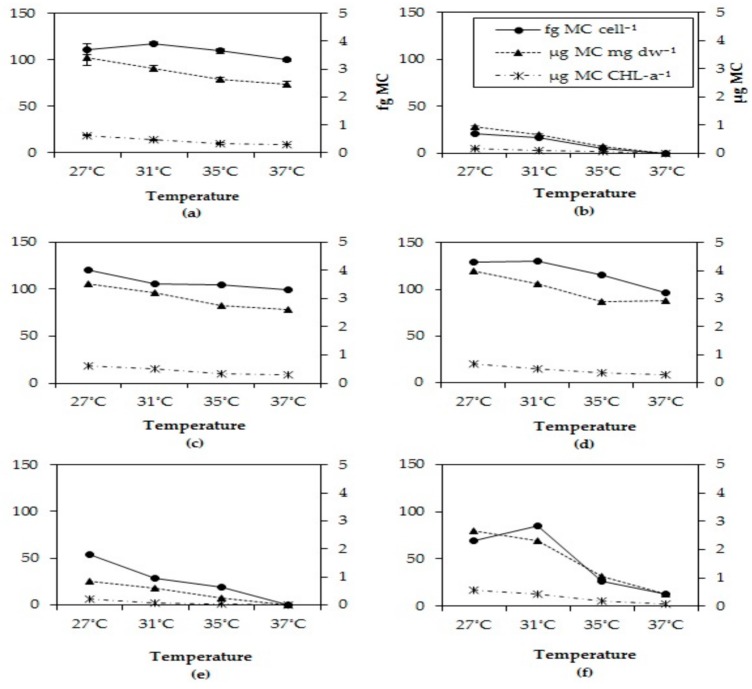
Total MC cell quota, total MC per unit chlorophyll-a and total MC per mg dry-weight in six different *Microcystis* strains grown at four different temperatures (**a**) MBC1, (**b**) MBC2, (**c**) MBC3, (**d**) MBC4, (**e**) MDT, (**f**) MTV.

MC cell quota dropped in all strains after six days at the two highest incubations temperatures tested (Figure 4). This pattern was observed for all three biomass indicators, MC content per cell, per dry-weight and per chlorophyll-a content. In each strain, the MC quota for all three biomass indicators significantly dropped at higher temperatures (Appendix D, Table A2)

## 3. Discussion

Our results support the hypothesis that elevated temperature may directly promote growth of tropical *Microcystis* but the temperature effect is strain dependent. All strains yielded higher biomass in terms of chlorophyll-a concentration and dry-weight at 31 °C compared to 27 °C and then either stabilised, slightly increased or declined with higher temperature. Growth rates were comparable to those found in the literature for *Microcystis* (Appendix E, Table A3). In general, growth rates showed optima around and above 31 °C that yielded an overall average optimum growth temperature of 34.3 °C (±2.3 °C) for the six strains and three endpoints used. This optimum growth temperature is somewhat higher than usually found in *Microcystis* (Appendix E, Table A3) but Thomas and Litchman [41] reported a similar optimum of 34.1 °C in one of their *M. aeruginosa* strains (Bear AC-02), Geada et al. [42] found the highest growth of *M. aeruginosa* at 35 °C and Mowe et al. [28] at 36 °C. For some of the strains we have tested the optimum growth rate might even be above 37 °C. That optima are higher than the temperatures these species may encounter in nature is regularly observed and explained from the asymmetry in the growth-temperature reaction norm, where growth rates decline faster beyond the optimum temperature than below it [41]. 

Our *Microcystis* strains were selected from sites that had water temperatures between 33.1 °C and 37.7 °C. The single strain that could not survive prolonged periods at 37 °C was strain MDT selected from the DauTieng reservoir that experienced a temperature of 33.5 °C at the moment of sampling. The average temperature in this reservoir was 31.05 °C with the maximal and minimal temperatures of 34 °C and 27.5 °C, respectively [38]. This indicates that the *Microcystis* strain MDT may have never been experienced at temperatures of 35 °C and 37 °C in the field as in our experiment. The highest temperature level 37 °C in our experiment, therefore, was too high for the *Microcystis* strain MDT to survive. Hence, five out of our six strains were able to grow at 37 °C. Likewise, in the literature considerable variability in the upper temperature limit *Microcystis* may tolerate is reported; four out of five tropical *Microcytis* were able to grow at 36 °C [28], one out of four *Microcystis* was able to grow at 35 °C but not at 40 °C, while another *Microcystis* even grew up to 40 °C [42]. Evidently, several *Microcystis* strains are well adapted to thrive under high temperature.

In the Southern region of Vietnam, the mean annual temperature increases from around 27 °C in January to above 30 °C in April; the warming temperature in April in recent years was contributed by heat waves in the Indochina peninsula [39]. This is in line with the prediction that the Mekong Delta will be one of the regions that will be most extremely influenced by climate change [31,32]. Particularly, dry season rainfall is projected to decrease [43], leading to a drier and longer dry season with heat waves pushing water temperatures up to 37 °C. This condition may be an appropriate recipe for more frequent and larger blooms of tropical *Microcystis* strains in the Southern region of Vietnam. 

More blooms or higher biomass, however does not necessarily imply higher health risk. Our results evidently show that in all strains total MC concentrations declined, toxicity expressed as MC-LR equivalents declined and that toxin quota declined with elevated temperatures. Similarly, [*D*-Leu^1^]-MC-LR concentrations in *M. aeruginosa* grown at 26 °C, 28 °C, 30 °C and 35 °C were 950, 500, 365 and 100 µg L^−1^, respectively, which was due to lower cell quota [44]. In two other *M. aeruginosa* strains total MC concentrations declined from 178 µg L^−1^ and 79 µg L^−1^ in cultures reared at 20 °C and 25 °C, respectively to 3 and to 2 µg L^−1^ in cultures at 35 °C [30]. Also in these strains MC quota dropped sharply with increasing temperature [30]. Lower MC quota at higher temperatures seems a more general pattern, because MC cell quota were lower in *M. aeruginosa* grown at 29 °C than at 26 °C [26], lower at 30 °C than at 20 °C [27] and were lower in five tropical *Microcystis* species grown at 36 °C than at lower temperatures [28]. A possible explanation for lower MC cell quota at higher temperatures is that MC may be a protectant against oxidative stress by binding to proteins [45]. Inasmuch as higher temperatures may increase oxidative stress in *Microcystis*, lower MC cell quota may be the results of binding of MCs to proteins [26]. The hot methanol extraction we have used will not liberate these bound MCs, which makes follow up research including the bound MC pool a logical step. For risk assessment, however, it remains to be determined if the rather irreversible thioether bond between the Mdha methylene group of MC and the thiol group of cysteine [46] can be broken easily.

The hot methanol (MeOH) extraction in our study uses 75% MeOH, which is far above the recommended >40% to avoid adsorption to laboratory tools when working with MC-containing solutions [47]. Lack of or acceptable loss was also confirmed by recovery determinations using spiked cyanobacterial matrix for the MC variants [48]. In addition to MCs, NOD was also included in the analysis. Low and trace amounts of NOD were found in the three most toxic strains. These strains were isolated from brackish water but no *Nodularia*—the most common NOD producer—was present in these waters. Recently, more reports of NOD from habitats that lacked *Nodularia* have appeared, including from lakes that were dominated by *Microcystis* [49,50]. Meanwhile, NOD has been shown being produced by *Nostoc* endosymbionts of Macrozamia [51], by free-living Nostoc from Brazil [52] and in a novel species *Iningainema pulvinus* that was isolated from a freshwater wetland spring [53].

**Figure A5:**
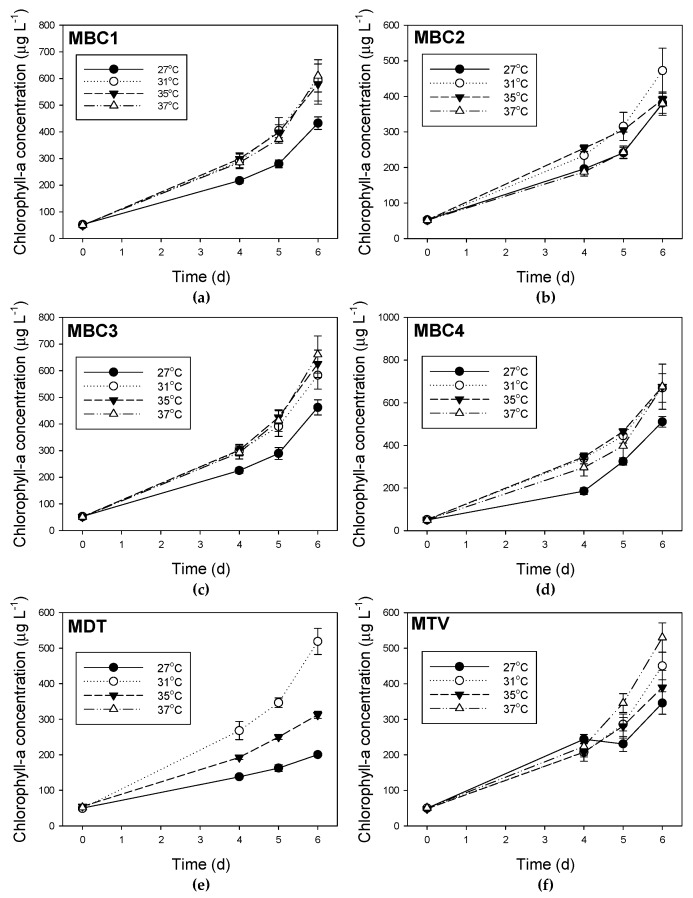
Course of chlorophyll-a concentrations for six *Microcystis* strains (**a**) MBC1, (**b**) MBC2, (**c**) MBC3, (**d**) MBC4, (**e**) MDT and (**f**) MTV grown at four different temperatures. Error bars indicate 1 SD.

**Figure A6:**
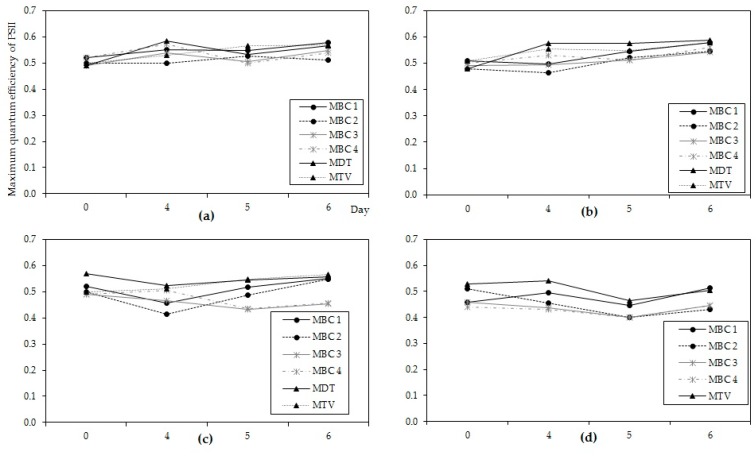
The maximum quantum efficiency of PS II in six *Microcystis* strains measuring at the start and on days 4, 5 & 6 at (**a**) 27 °C, (**b**) 31 °C, (**c**) 35 °C and (**d**) 37 °C. Error bars indicate 1 SD.

The observed decline of total MC concentrations with higher temperatures differed in strains with deviating MC profiles. In the strains with predominantly MC-LR (MBC1,3,4), total MC concentrations were 27% (±3%) lower at 35 °C and 35% (±6%) lower at 37 °C than 27 °C but in strains with MC-RR as dominant variant total MC concentrations were 67% (±5%) lower at 35 °C and 94% (±11%) lower at 37 °C than at 27 °C. Hence, the response to temperature is strain dependent and seems more pronounced in strains producing less toxic MC variants (dmMC-RR, MC-RR). Since arginine (R) contains three more bound nitrogen atoms than leucine (L), a stronger decrease in MC-RR might be caused by lower arginine cell content under enhanced nitrogen limitation [54]. However, we have no indications that there would have been strong N limitation in the higher temperatures, whilst not at lower temperature. Nitrogen limitation will impair amino acid synthesis [54] but also leading to chlorosis [55] and reduced growth rates [56]. All cultures expressed exponential growth, except of course MDT at 37 °C, were quite greenish from appearance and no loss of pigments was indicated from PHYTO-PAM analyses (Appendix G, Figure A5 and Figure A6).

**Figure A7:**
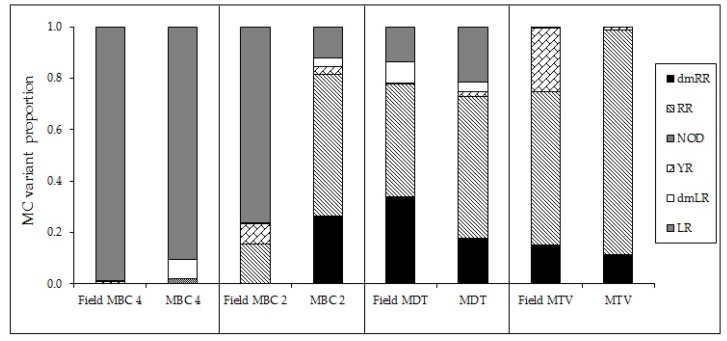
Proportions of different MC-variants and NOD in four field samples and isolated strains collected in the same position.

In addition to clearly declined absolute amounts and cell quota of MCs with elevated temperatures, also the MC profiles were changed. In five out of six strains the proportion of the most toxic variant MC-LR increased with higher temperatures, which has also been observed by [26]. The response of MC-RR was less clear and either its proportion increased with temperature or decreased, again illustrating strain specific responses to increased temperature. *Dolichospermum* sp. showed relatively more MC-RR at higher temperatures [57]. A similar response was found by Maliaka et al. [58] who observed that the share of MC-RR in the total MC-pool of incubated lake seston at 20 °C was 47% (±5)% but increased to on average 62% (±3)% at 25 °C and 66% (±8)% at 30 °C. In contrast, Amé and Wunderlin [59] found a higher proportion of MC-LR at higher temperature (28 °C) than at lower temperature (20 °C) and the opposite for MC-RR. These authors also noted higher MC-LR cell quota and lower MC-RR cell quota, also when expressed per protein biomass [59]. Those studies show that the MC-profile in *Microcystis* may be changed with elevated temperature, which could be caused by altered availability of certain amino acids [54] but such hypothesis needs further investigation. In the studies with incubated field samples [58,59], however, also competitive replacement of strains varying in toxin profile could have occurred. In our experiment, the altered MC profiles can be ascribed to changes in the production and/or fate of MC variants produced by each isolate. Whether these isolates are fully representative for the actual field locations is difficult to decipher. Nonetheless, the MC profiles of field samples collected at the same sites where the strains, MBC4, MDT and MTV were isolated showed high similarity to the MC profiles of the corresponding strains (Appendix H, Figure A7). The profile of MBC2, however, deviated from the field sample and thus this isolate might not have been the main toxin producer during the bloom event. Field data (MC profiles) from locations MBC1 and MBC3 are not available.

In field populations, higher temperatures may promote growth rates of toxigenic *Microcystis* cells leading to a larger proportion of toxic cells [19]. The different MC profiles and MC quota as well as strain specific response to higher temperatures make it difficult to predict what will be the expected toxicity, also since “chance is the pacemaker of evolution of toxin production” [60]. However, there is little doubt that aggravated eutrophication will lead to more cyanobacterial biomass and since the MC concentration is determined by the biomass of toxigenic strains and their toxin quota, despite MC quota seem to decline with higher temperatures, elevated in situ MC concentrations may occur [30,58]. Also in Southern Vietnam surface accumulations of cyanobacteria are occurring, which is an important biomass concentrating event especially during heat wave episodes, in which extremely high MC concentrations have been measured [61]. i.e. MC concentration of field samples collected in TraVinh at 33.1 °C was 4033 µg g^−1^ dry-weight that was 1.5; 1.8; 3.9 and 9.6 times higher than that of the *Microcystis* strain MTV grown at 27 °C, 31 °C, 35 °C and 37 °C, respectively. Hence, although cells may become less toxic in a near future Southern Vietnam, the expected surface accumulations and increased biomass under predicted climate change, urge for measures to reduce the cyanobacterial biomass drastically.

## 4. Conclusions

Temperature had significant effects on growth rates and MC production of tropical *Microcystis* strains. Raising temperatures above 27 °C lead to increased growth rates but decreased MC production. Five out of six strains survived up to 37 °C. Blooms of tropical *Microcystis* may become more frequent in Southern Vietnam under warming climate, while *Microcystis* strains may become less toxic.

## 5. Materials and Methods 

### 5.1. Microcystis Strains

**Figure 5:**
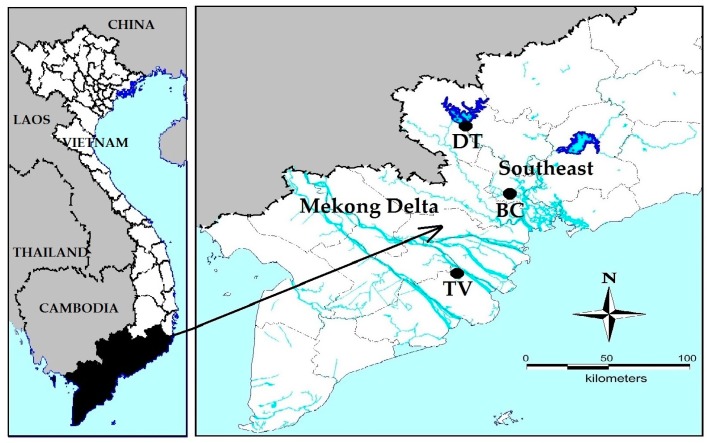
Location of the sampling sites in South Vietnam. BC = four fishponds in BinhChanh district (HoChiMinh city), where strains MBC1, MBC2, MBC3 and MBC4 were isolated. DT = DauTieng reservoir (TayNinh province) where strain MDT was collected. TV = fish pond in Mekong delta (TraVinh province), where strain MTV was isolated.

Six strains of *Microcystis* were collected and isolated during bloom events, in which 4 strains were from several fish ponds in BinhChanh district, HoChiMinh city (so called MBC1, MBC2, MBC3 and MBC4), one strain was from DauTieng reservoir with a surface area of 270 km^2^ in DauTieng district, TayNinh province (so called MDT) and the last strain was from a fish pond in Mekong delta, TraVinh province (so called MTV), Vietnam (Figure 5). At each collecting site, temperature, salinity and pH were measured by a portable WTW 340i meter (WTW, Weilheim, Germany). Chlorophyll-a was measured with an AlgaeTorch (bbe Moldaenke GmbH, Schwentinental, Germany, see Appendix F for calibration). Samples from the fishponds were measured in a bucket after dilution of collected scum material within tap water to remain within the advised measuring range for the AlgaeTorch. Scum samples and water samples were transported to the laboratory for further analyse. Nutrients in the field water samples were analysed colorimetrically with a spectrophotometer (Hach R/2010) using the following APHA (2005) [62] methods: nitrate 4500NO_3_^−^, ammonium 4500NH_4_^+^, total nitrogen (TN) Kjeldahl 4500N, phosphate and total phosphorus (TP) 4500P. The detection limits of the equipment for these parameters were 0.02 mg L^−1^ (nitrate), 0.04 mg L^−1^ (ammonium), 0.06 mg L^−1^ (TN Kjeldahl) and 0.05 mg L^−1^ for both TP and phosphate.

In the laboratory, single *Microcystis* cells or colonies were picked out of the collected scum material by the micropipette-washing method [63]. These *Microcystis* strains were grown in small glass tubes with a few mL modified WC medium (Woods Hole modified CHU10-medium, [64]) for several months at 25 °C, under a 14:10 h light/dark cycle at a light intensity of 70 µmol photon.m^−2^ s^−1^. When strains reached a greenish appearance, they were transferred into 50 mL Erlenmeyer flasks and subsequently into 250 mL flasks.

### 5.2. Growth Experiment 

The six *Microcystis* strains were first acclimatised to the experimental conditions (see below) for a week. Then they were inoculated in 300-mL Erlenmeyer flasks that contained 200 mL WC medium. Flasks were closed with a cellulose plug. The initial *Microcystis* concentration in the flasks was 52 ± 3 µg chlorophyll-a L^−1^. For each strain, triplicate flasks were placed for 6 days at 27 °C (low), 31 °C (medium), 35 °C (high) and 37 °C (extreme high) in Sanyo Gallenkamp orbital incubators (MLR-351H, SANYO Electric Co., Ltd., Osaka, Japan). The flasks were illuminated at a maximum light intensity of ~130 μmol quanta m^−2^ s^−1^ in a 14 h:10 h light dark cycle. Flasks were shaken manually twice every day.

Subsamples of 2.5 mL were taken at the start and after 4th, 5th and 6th day of incubation and analysed on chlorophyll-a concentration and Photosystem-II (PSII) efficiency using a PHYTO-PAM phytoplankton analyser (HeinzWalz GmbH, Effeltrich, Germany) and on particle- and bio volume concentrations using a CASY counter (Casy TTC, Schärfe System, GmBh, Reutlingen, Germany). At the end of the incubation additionally 15 mL and 25 mL culture material was filtered over two separate glass-fibre filters (Whatman GF/C), of which one was stored at −20 °C for MC analysis (see Section 5.4). The other one that was weighed before filtration was put in an oven at 60 °C for 48 h and subsequently the dry-weight was measured on a 0.01-mg resolution balance (Sartorius R 160P, Göttingen, Germany).

### 5.3. Microcystin (MC) Analysis

The Frozen filters stored at −20 °C were transferred to 8 mL glass tubes and dried for two hours in a freeze-drier (Alpha 1-2 LD, Martin Christ Gefriertrocknungsanlagen GmbH, Osterode am Harz, Germany). The filters were extracted three times at 60 °C in 2.5 mL of 75% methanol-25% Millipore water (*v/v*). The extracts were then dried in the Speedvac (Savant SPD121P, Thermo Scientific, Waltham, MA, USA) and subsequently reconstituted in 900 μL 100% methanol. The reconstituted samples were transferred to 2 mL Eppendorf vials with a cellulose-acetate filter (0.2 μm, Grace Davison Discovery Sciences, Deerfield, IL, USA) and centrifuged for 5 min at 16,000× *g* (VWR Galaxy 16DH, VWR International, Buffalo Grove, IL, USA). Filtrates were then transferred to amber glass vials for LC-MS/MS analysis. 

Concentrations of eight MC variants (dm-7-MC-RR, MC-RR, MC-YR, dm-7-MC-LR, MC-LR, MC-LY, MC-LW and MC-LF) and nodularin (NOD) were determined by LC-MS/MS as described in [30,48]. LC-MS/MS analysis was performed on an Agilent 1200 LC and an Agilent 6410A QQQ (Agilent Technologies, Santa Clara, CA, USA). The MCs were separated on an Agilent Eclipse 4.6 × 150 mm, 5-μm column. Hereto, 10 μL sample was injected; the flow rate was 0.5 mL/min; the column temperature was 40 °C. Eluents were Millipore water with 0.1% formic acid (*v/v*, Eluent A) and acetonitrile with 0.1% formic acid (*v/v*, Eluent B) that were run using an elution program of 0–2 min 30% B, 6–12 min 90% B, with a linear increase of B between 2 and 6 min and a 5-min post run at 30% B. Detailed information on MS/MS settings for each MC can be found in [65]; information on recovery, repeatability, limit of detection and limit of quantification of the analysis is given in [48]. MCs were quantified against certified standards that were obtained from DHI LAB Products (Hørsholm, Denmark). 

### 5.4. Data Analysis

Growth rates (µ, d^−1^) based on the increase of chlorophyll-a concentrations over time as well as over the increase in particle concentration over time, were determined using the equation:

(1) $$µ=\frac{\ln\left(Wt\right)-\ln\left(Wo\right)}{t}$$

in which *Wt* is the chlorophyll-a- or particle concentration of the *Microcystis* culture at sampling time *t*, *Wo* is the initial chlorophyll-a- or particle concentration and *t* is the elapsed time (days) between *Wt* and *Wo*.

Chlorophyll-a concentrations, particle concentrations and dry-weights determined at the end of the experiment were analysed each by two-way ANOVA with strain and temperature as factors. Tukey post-hoc comparison was used to identify differences amongst means. Normality was tested using a Shapiro-Wilk test, whereas homogeneity of variance was tested by Levene’s Equal Variance Test. Because data transformation could not solve violations of these ANOVA requirements, growth rates and total MC concentrations were analysed per strain by running one-way ANOVAs or Kruskal-Wallis one-way ANOVA on Ranks that were followed by Holm-Sidak or Tukey post-hoc comparisons, respectively. All statistical analyses were performed using the program SigmaPlot (version 13.0; Systat Software Inc., San Jose, CA, USA).

## Figures and Tables

**Table 1:** Water temperature (°C), salinity (‰), pH, total nitrogen (TN), total phosphorus (TP), dissolved nutrients and chlorophyll-a (CHLa) concentrations in six water bodies from which *Microcystis* strains were isolated (MBC1, MBC2, MBC3, MBC4, MDT, MTV).

Strain	Temp. (°C)	Salinity (‰)	pH	TN (mg/L)	TP (mg/L)	N-NH_4_ (mg/L)	N-NO_3_ (mg/L)	P-PO_4_ (mg/L)	CHLa (µg/L)
MBC1	37.5	7.4	9.89	9.13	0.25	0.57	BDL	BDL	1480
MBC2	37.4	7.5	9.77	9.00	0.33	0.67	BDL	BDL	1520
MBC3	37.4	7.4	9.79	9.36	0.30	0.46	BDL	BDL	1437
MBC4	37.7	7.6	9.66	16.91	1.52	1.02	0.04	0.19	5100
MDT	33.5	0.0	9.82	3.98	0.59	0.39	0.14	0.10	169
MTV	33.1	0.5	9.58	19.5	1.75	1.14	0.28	BDL	4352
BDL, below detection limit									

**Table 2:** Mean growth rates (d^−1^) based chlorophyll-a, particle concentrations and bio volume concentrations of six *Microcystis* strains grown at four different temperatures. Standard deviations are given in parentheses (*n* = 3), while different letters for each strain (per row) indicate significant differences (Tukey test, *p* < 0.050).

**Strain**	**Chlorophyll-a Based Growth Rates**			
	**27 °C**	**31 °C**	**35 °C**	**37 °C**
MBC1	0.35 (0.01)^A^	0.42 (0.03)^B^	0.42 (0.03)^B^	0.41 (0.02)^B^
MBC2	0.32 (0.02)^A^	0.37 (0.03)^B^	0.36 (0.03)^B^	0.32 (0.02)^A^
MBC3	0.35 (0.02)^A^	0.42 (0.02)^B^	0.42 (0.02)^B^	0.43 (0.02)^B^
MBC4	0.36 (0.03)^A^	0.44 (0.03)^B^	0.45 (0.02)^B^	0.43 (0.03)^B^
MDT	0.24 (0.01)^A^	0.40 (0.02)^B^	0.30 (0.01)^C^	---
MTV	0.34 (0.04)^A^	0.35 (0.02)^A^	0.36 (0.03)^A^	0.38 (0.03)^A^
**Strain**	**Particle Concentration Based Growth Rates**			
	**27 °C**	**31 °C**	**35 °C**	**37 °C**
MBC1	0.28 (0.03)^A^	0.33 (0.04)^B^	0.41 (0.04)^C^	0.35 (0.02)^B^
MBC2	0.33 (0.02)^A^	0.37 (0.04)^A^	0.34 (0.03)^A^	0.35 (0.02)^A^
MBC3	0.33 (0.03)^A^	0.34 (0.04)^A^	0.42 (0.03)^B^	0.39 (0.02)^B^
MBC4	0.35 (0.06)^AB^	0.33 (0.04)^A^	0.40 (0.03)^C^	0.39 (0.06)^BC^
MDT	0.18 (0.03)^A^	0.37 (0.01)^B^	0.27 (0.01)^C^	---
MTV	0.31 (0.04)^AB^	0.31 (0.03)^A^	0.44 (0.04)^C^	0.25 (0.06)^B^
**Strain**	**Biovolume Concentration Based Growth Rates**			
	**27 °C**	**31 °C**	**35 °C**	**37 °C**
MBC1	0.31 (0.04)^A^	0.34 (0.06)^A^	0.44 (0.08)^B^	0.38 (0.05)^AB^
MBC2	0.34 (0.06)^A^	0.45 (0.11)^AB^	0.54 (0.09)^B^	0.19 (0.03)^A^
MBC3	0.41 (0.06)^A^	0.42 (0.07)^A^	0.46 (0.04)^A^	0.23 (0.04)^B^
MBC4	0.25 (0.05)^A^	0.29 (0.04)^A^	0.38 (0.04)^B^	0.45 (0.06)^C^
MDT	0.39 (0.03)^AB^	0.41 (0.05)^A^	0.30 (0.02)^B^	---
MTV	0.27 (0.07)^A^	0.38 (0.07)^A^	0.36 (0.07)^A^	0.40 (0.06)^A^

**Table 3:** Results of separate one-way ANOVA on total microcystin concentrations in six different *Microcystis* strains cultured for six days at four different temperatures. Similar letters per column indicate homogeneous groups that are not different at the 95% level (Holm-Sidak post hoc test, except for strain MTV, Tukey test).

Temp.	*Microcystis* Strains					
	MBC1	MBC2	MBC3	MBC4	MDT	MTV ^#^
27 °C	A	A	A	A	A	A
31 °C	A	B	A	A	A	AB
35 °C	B	C	B	B	B	AB
37 °C	B	D	B	B	C	B
*F*_3,11_ value	17.6	94.4	14.7	24.4	149.1	*H*_3_ = 9.5
*p-*value	*p* < 0.001	*p* < 0.001	*p* = 0.001	*p* < 0.001	*p* < 0.001	*p =* 0.024
Normality	*p* = 0.555	*p* = 0.627	*p* = 0.420	*p* = 0.374	*p* = 0.114	*p* = 0.439
Equal Var.	*p* = 0.634	*p* = 0.323	*p* = 0.863	*p* = 0.307	*p* = 0.455	*p* < 0.050

**^#^** MC data of strain MTV violated assumption of equal variance that could not be solved by data transformation. Non-parametric Kruskal-Wallis One Way Analysis of Variance on Ranks was run instead.

**Figure A4:**
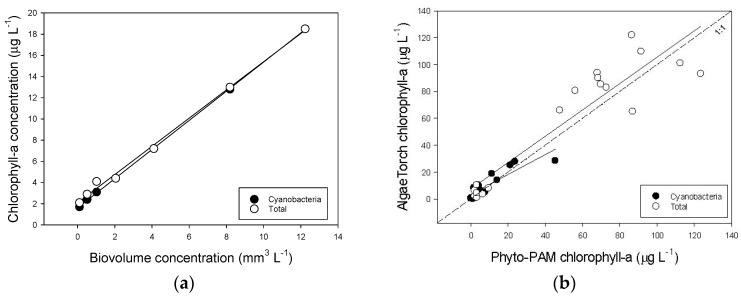
(**a**) Relationship between the bio volume concentration of a laboratory culture of the cyanobacterium *Anabaena cylindrica* PCC7122 and the cyanobacterial—and total chlorophyll-a concentrations determined with the AlgaeTorch. (**b**) Relationships between the cyanobacterial—and total chlorophyll-a concentrations determined with the Phyto-PAM and with the AlgaeTorch.

## Appendix A

Total microcystin concentrations (MCs) were normalized against the mean MC concentration in the 27 °C incubations of each strain. The normalized MC concentrations were significantly reduced at elevated temperatures (One-way ANIOVA on ranks; *H*_3_ = 53.426; *p* < 0.001) where a Tukey post hoc comparison test revealed two homogeneous groups: (1) the 27 °C and 31 °C treatments and (2) the 35 °C and 37 °C treatments (Figure A1).

## Appendix B

Different MC variants may differ considerably in their toxicity [66,67]. The contribution of each variant to the overall MC toxicity of a sample was determined by assigning a toxicity factor to each variant (see Table 1 in [67]) and by multiplying variant concentrations by these toxicity factors. These toxicity factors for the variants determined in this study (dm-7-MC-RR, MC-RR, MC-YR, dm-7-MC-LR) were estimated from the average i.p. LD_50_ values to mice as given in [66]. Toxicity was then expressed in MC-LR equivalents. Two groups could be distinguished: (1) the more toxic strains MBC1, MBC3 and MBC4 (dominated by the variant MC-LR) and (2) the less toxic strains MBC2, MDT and MTV that had pre-dominantly MC-RR (Figure A2). In both groups, toxicity was significantly reduced at higher temperatures; *F*_3,11_ = 11.6; *p* = 0.003 for the more toxic group and *F*_3,11_ = 19.8; *p* < 0.001 for the less toxic group. In both groups two homogeneous groups of MC-LR equivalents were detected: (1) highest at 27 °C and 31 °C, (2) significantly lower at 35 °C and 37 °C (Holm-Sidak post hoc comparison).

## Appendix C

Proportions of different MC-variants and NOD detected in the six strains from Southern Vietnam after six days growth at four different temperatures (Figure A3). Statistical evaluation for the two most abundant MC-variants (MC-LR, MC-RR) was done by separate one-way ANOVAs per strain followed by Holm-Sidak post hoc comparison test to identify differences, unless stated otherwise (Table A1).

**Table A1:** Results of separate one-way ANOVA on the proportions of the most abundant MC variants (MC-LR, MC-RR) in six different *Microcystis* strains cultured for six days at four different temperatures. Similar letters per column indicate homogeneous groups that are not different at the 95% level (Holm-Sidak post hoc test, except for strain MTV, Tukey test).

**MC-LR**	***Microcystis* Strains**					
**Temp.**	**MBC1**	**MBC2**	**MBC3**	**MBC4**	**MDT**	**MTV**
27 °C	A	A	A	A	A	A
31 °C	B	A	B	B	AB	A
35 °C	B	A	B	B	B	A
37 °C	C	B	C	C	---	A
*F*_3,11_ value	333.2	187.8	290.1	153.6	6.84	0.75
*p-*value	*p* < 0.001	*p* < 0.001	*p* = 0.001	*p* < 0.001	*p* = 0.028	*p =* 0.551
Normality	*p* = 0.759	*p* = 0.085	*p* = 0.605	*p* = 0.219	*p* = 0.967	*p* = 0.217
Equal Var.	*p* = 0.256	*p* = 0.063	*p* = 0.605	*p* = 0.654	*p* = 0.294	*p* = 0.480
**MC-RR**	***Microcystis* Strains**					
**Temp.**	**MBC1**	**MBC2 ^@^**	**MBC3**	**MBC4**	**MDT**	**MTV ^#^**
27 °C	---	A	---	---	A	A
31 °C	---	A	---	---	B	AB
35 °C	---	B	---	---	B	AB
37 °C	---	---	---	---	---	B
*F*_3,11_ value	---	50.70	---	---	55.65	*H_3_* = 10.4
*p-*value	---	*p* < 0.001	---	---	*p* = 0.028	*p =* 0.015
Normality	---	*p* = 0.168	---	---	*p* = 0.300	*p* < 0.050
Equal Var.	---	*p* = 0.190	---	---	*p* = 0.205	*---*

^@^ MBC2 was analysed omitting the zeros in the 37 °C treatment to avoid non-normality. **^#^** MC-RR data for strain MTV violated assumption of normal distribution that could not be solved by data transformation. Non-parametric Kruskal-Wallis One Way Analysis of Variance on Ranks was run instead.

## Appendix D

**Table A2:** Results of separate one-way ANOVA on the MC quota based on three biomass estimates (cells, chlorophyll-a [CHLa], dry-weight [DW]) in six different *Microcystis* strains cultured for six days at four different temperatures. Similar letters per column indicate homogeneous groups that are not different at the 95% level (Holm-Sidak post hoc test, except for strain MDT indicated by #, Tukey test).

**fg MC cell^−1^**	***Microcystis* Strains**					
**Temp.**	**MBC1**	**MBC2**	**MBC3**	**MBC4**	**MDT**	**MTV**
27 °C	A	A	A	A	A	A
31 °C	A	B	AB	A	B	B
35 °C	A	C	AB	AB	C	C
37 °C	B	D	B	B	D	D
*F*_3,11_ value	13.76	481.3	5.44	7.26	528.5	535.3
*p-*value	*p* = 0.002	*p* < 0.001	*p* = 0.025	*p* = 0.011	*p* < 0.001	*p <* 0.001
Normality	*p* = 0.746	*p* = 0.197	*p* = 0.256	*p* = 0.136	*p* = 0.211	*p* = 0.597
Equal Var.	*p* = 0.289	*p* = 0.129	*p* = 0.928	*p* = 0.754	*p* = 0.659	*p* = 0.211
**µg MC µg CHLa^−1^**	***Microcystis* Strains**					
**Temp.**	**MBC1**	**MBC2**	**MBC3**	**MBC4**	**MDT ^#^**	**MTV**
27 °C	A	A	A	A	A	A
31 °C	B	B	B	B	AB	B
35 °C	C	C	C	C	AB	C
37 °C	D	D	D	D	B	D
*F*_3,11_ value	137.5	457.5	274.4	250.2	*H_3_ =* 10.5	1167.7
*p-*value	*p* < 0.001	*p* < 0.001	*p* < 0.001	*p* < 0.001	*p* = 0.015	*p* < 0.001
Normality	*p* = 0.322	*p* = 0.235	*p* = 0.755	*p* = 0.714	*p* = 0.159	*p* = 0.443
Equal Var.	*p* = 0.225	*p* = 0.135	*p* = 0.768	*p* = 0.079	*p* < 0.050	*p* = 0.241
**µg MC mg DW^−1^**	***Microcystis* Strains**					
**Temp.**	**MBC1**	**MBC2**	**MBC3**	**MBC4**	**MDT**	**MTV**
27 °C	A	A	A	A	A	A
31 °C	B	B	AB	AB	AB	B
35 °C	C	C	BC	B	AB	C
37 °C	C	D	C	B	B	D
*F*_3,11_ value	20.77	396.0	11.11	9.77	103.2	151.9
*p-*value	*p* < 0.001	*p* < 0.001	*p* = 0.003	*p* = 0.005	*p* < 0.001	*p* < 0.001
Normality	*p* = 0.741	*p* = 0.269	*p* = 0.915	*p* = 0.091	*p* = 0730	*p* = 0.799
Equal Var.	*p* = 0.084	*p* = 0.685	*p* = 0.430	*p* = 0.626	*p* = 0.561	*p* = 0.390

^#^ Non-parametric Kruskal-Wallis One Way Analysis of Variance on Ranks was run.

## Appendix E

A selection of studies from the literature in which growth of *Microcystis* was evaluated at different temperatures reveals that the average temperature of optimum growth is above 30 °C and in some studies even 35 °C (Table A2). Maximum growth rates ranged between 0.2 and 1.6 d^−1^ (Table A2).

**Table A3:** Literature data for temperature range tested, optimum growth temperature and growth rate at optimum for *Microcystis*.

Species	*T* Range	T Opt	µ_max_ (d^−1^)	Reference
*M. aeruginosa*	20–35	30	0.45	[68]
*M. aeruginosa*	20–35	32	1.6	[56]
*M. aeruginosa*	16–36	32	0.81	[25]
*M. aeruginosa*	15–30	25	0.19	[69]
*M. aeruginosa*	5–25	25	0.36	[70]
*M. aeruginosa*	10–30	27.5	0.8	[71]
*M. aeruginosa*	10–45	35	1.06	[72]
*M. aeruginosa*	20–35	32.5	1.12	[73]
*M. aeruginosa*	20–35	30	0.94	[73]
*M. flos-aquae*	27–36	30	0.81	[28]
*M. ichthyoblabe*	27–36	33	0.94	[28]
*M. ichthyoblabe*	27–36	33	0.77	[28]
*M. aeruginosa*	27–36	36	0.65	[28]
*M. viridis*	27–36	30	0.49	[28]
*M. aeruginosa*	18–30	30	0.4	[74]
*M. aeruginosa*	15–40	28.3	0.38	[39]
*M. aeruginosa*	15–40	28.8	0.41	[39]
*M. aeruginosa*	15–40	34.1	0.62	[39]
*M. aeruginosa*	15–40	28	0.29	[39]
*M. aeruginosa*	15–35	30	0.28	[39]
*M. aeruginosa*	15–40	35	1.02	[39]
	Average	30.7	0.69	
	SD	3.1	0.35	

## Appendix F

The AlgaeTorch was tested in the laboratory using a culture of *Anabaena cylindrica* PCC7122, of which the bio volume concentration was determined using the CASY counter. Different portions of culture material were diluted in 2 L medium and the cyanobacterial- and total chlorophyll-a concentrations were measured with the AlgaeTorch. This yielded: cyanobacterial chlorophyll-a = 1.6178 + 1.3745 × Biovolume (*R*²= 0.999); total chlorophyll-a = 2.0946 + 1.3306 × Biovolume (*R*² = 0.997) (Figure A4a). Subsequently, the AlgaeTorch (AT) was taken into the field where cyanobacterial—and total chlorophyll-a concentration were measured while a sample of the water was also analysed on a PHYTO-PAM (PP) phytoplankton analyser (PHYTO-ED, Heinz Walz GmbH, Effeltrich, Germany) that had been calibrated against the Dutch standard [75], which is a hot ethanol extraction based on [76]. This yielded acceptable relations for cyanobacterial chlorophyll-a: AT = 3.3067 + 0.7399 × PP (*R*² = 0.784) and for total chlorophyll-a: AT = 7.2522 + 0.9816 × PP (*R*² = 0.855) (Figure A4b).
